# Needle-scalpel therapy inhibits the apoptosis of nucleus pulposus cells via the SDF-1/CXCR4 axis in a rat degenerative cervical intervertebral disc model

**DOI:** 10.18632/aging.205959

**Published:** 2024-06-20

**Authors:** Wenlong Yang, Muqing Liu, Qinran Sun, Lei Liu, Wenqing Wu, Fangming Liu, Zhizhen Liu

**Affiliations:** 1 Department of Rehabilitation Medicine, The First Affiliated Hospital of Shandong First Medical University and Shandong Provincial Qianfoshan Hospital, Shandong Institute of Anesthesia and Respiratory Critical Medicine, Jinan, Shandong, China; 2 School of Acupuncture-Tuina, Shandong University of Traditional Chinese Medicine, Shandong, China

**Keywords:** Needle-scalpel, intervertebral disc degeneration, apoptosis of nucleus pulposus cells, SDF-1/CXCR4 axis

## Abstract

As a common disease, cervical spondylosis (CS) results from the degeneration of the cervical intervertebral disc. However, there are still no effective clinical strategies for the treatment of this disease. Needle-scalpel (Ns), a therapy guided by traditional Chinese medicine theory, alleviates intervertebral disc degradation and is widely used in the clinic to treat CS. Stromal cell-derived factor-1 (SDF-1) and its receptor CXC receptor 4 (CXCR4) in nucleus pulposus cells play an important role in CS onset and development. This study aimed to explore whether Ns can relieve pain and regulate the SDF-1/CXCR4 axis in nucleus pulposus cells to inhibit apoptosis, thereby delaying cervical intervertebral disc degradation in a rat model of CS. It was found that the Ns-treated groups exhibited higher mechanical allodynia scores than the model group, and H&E staining, MRI, and scanning electron microscopy revealed that Ns therapy inhibited intervertebral disc degeneration. Additionally, Ns therapy significantly inhibited increases in the RNA and protein expression levels of SDF-1 and CXCR4. Furthermore, these treatments alleviated the apoptosis of nucleus pulposus cells, which manifested as a decline in the proportion of apoptotic nucleus pulposus cells and inhibition of the decrease in the levels of Bcl-2/Bax. These findings indicated that Ns mitigated CS-induced pain, inhibited the apoptosis of nucleus pulposus cells, and alleviated intervertebral disc degeneration in CS rats. These effects may be mediated by specifically regulating the SDF-1/CXCR4 signaling axis. Based on these findings, we conclude that Ns might serve as a promising therapy for the treatment of CS.

## INTRODUCTION

Cervical spondylosis (CS) represents a commonly diagnosed human disorder that generally results from intervertebral disc degeneration (IVDD). CS imposes substantial economic and health burdens on society and decreases patients’ quality of life [1–3]. The intervertebral disc mostly comprises the internal nucleus pulposus (NP), external annulus fibrosus, and endplate [4, 5]. The NP responds to various mechanical stresses [6], and NP cells (NPCs) in the NP tissue regulate the metabolism of the extracellular matrix (ECM), which is rich in type II collagen and resistant to spinal compressive loads [7]. Studies have shown that mechanical instability of the cervical spine increases apoptosis in NPCs, thereby reducing the generation of type II collagen in the ECM, ultimately decreasing the compressive strength of the intervertebral disc and inducing its degeneration [8–10]. Current therapeutic approaches for CS encompass pharmacological and nonpharmacological treatments, including surgery [11–15]. However, only surgery effectively alleviates pain in end-stage CS. With a rising prevalence, CS attracts increasing attention regarding the development of effective approaches for prevention and early treatment, especially nonsurgical strategies.

CS-induced neck pain represents an important outcome of disease progression [16]. Meanwhile, the Global Burden of Disease 2010 Study revealed neck pain as the fourth factor causing disability [17] and inducing psychosocial problems, especially stress-related issues such as depression, anxiety, and fear of movement [2], resulting in seriously declined quality of life. Consequently, CS treatment mostly involves pain alleviation and retardation of disease progression [18, 19]. Current guidelines for first-line treatment of CS recommend nonsteroidal anti-inflammatory drugs (NSAIDs), which, however, have severe adverse reactions, and their prolonged use is not recommended [20, 21]. Therefore, nonpharmacological and/or alternative therapeutic approaches are urgently needed in CS [22].

Needle-scalpel (Ns), a nonpharmacological intervention, is a popular traditional treatment method. Ns represents a biomechanical treatment considering modern anatomical features and applying a traditional Chinese medicine theory on the meridian nerve. Ns, integrating acupoint stimulation and local loosening, simultaneously confers the benefits of acupuncture needles and scalpels [23]. In the treatment of CS, Ns is used to remove the attached tissues. It protects the cervical intervertebral disc via adjustment of the mechanical features of the soft tissues around the cervical vertebra, relieving stress around the NP and delaying its degeneration [24–26]. Our previous study suggested that Ns therapy could inhibit IVDD by modulating the ECM collagen system and improving the altered structure of the NP [27]. Based on the significant effect of NPC apoptosis on IVDD [28, 29], it was hypothesized that Ns therapy inhibits NPC apoptosis in CS rats.

Stromal cell-derived factor-1 (SDF-1) is an extensively investigated chemokine. SDF-1 was first detected in bone marrow mesenchymal stem cells and induces multiple downstream signal transduction pathways via interaction with C-X-C motif chemokine receptor 4 (CXCR4), which then affects intervertebral disc internal ECM synthesis and degradation, stem cell chemotaxis, inflammatory chemotaxis, and vascular regeneration [30–33]. Additionally, it reportedly affects cell apoptosis and regulates the mechanical properties of the degenerated intervertebral disc [34–37]. Thus, this study explored the possible mechanism by which Ns therapy inhibits NPC apoptosis and relieves cervical IVDD. The data demonstrated that Ns therapy inhibited NPC apoptosis and relieved cervical disc degeneration, likely via the SDF-1/CXCR4 axis.

## MATERIALS AND METHODS

### Animals and groups

Healthy male clean-grade Sprague–Dawley rats (200 ± 20 g) provided by Jinan Pengyue Experimental Animal Breeding (Shandong, China; certificate no. SCXK Lu 2019–0003) underwent housing at 23°C ± 2°C under a 12-h photoperiod with ad libitum food and water. Studies involving animals followed the Guide for the Care and Use of Laboratory Animals released by the United States National Institutes of Health. The animal ethics committee of the First Affiliated Hospital of Shandong First Medical University and Shandong Provincial Qianfoshan Hospital approved the protocol of this study ((2019) no. S177).

During model establishment, 5 rats died of wound infection or unknown causes. Finally, 35 rats completed 3 months of modeling. CS model rats were then randomized to the Model (CS model), M+Ns (CS model + Ns treatment), and M+Ms (CS model + celecoxib medicaments treatment) groups. All treatments lasted 4 weeks.

### CS model establishment

Forty rats were used for CS model establishment as described in a previous study [27]. Briefly, following a 1-week adaptive housing, the animals underwent anesthesia by intraperitoneally injecting 3% pentobarbitone sodium at 30 mg/kg. After skin disinfection with Anerdian, a posterior cervical median incision exposed the tissue from the atlanto-occipital joint to the second thoracic vertebra. This was followed by a separation of fascia, platysma muscle, cervical trapezius, and rhomboideus, with muscle stripping to expose its deep layer. After fully exposing cervical vertebrae, the C2–C7 supraspinous and interspinous ligaments were snipped. Next, bilateral sacrospinal muscles and skin were sutured. Two random model animals underwent euthanasia 12 weeks following model establishment. C4/5 and C5/6 intervertebral discs underwent fixation. The sham group was treated by a sham operation, with only the skin along the nuchal incised and sutured.

**Figure 1:**
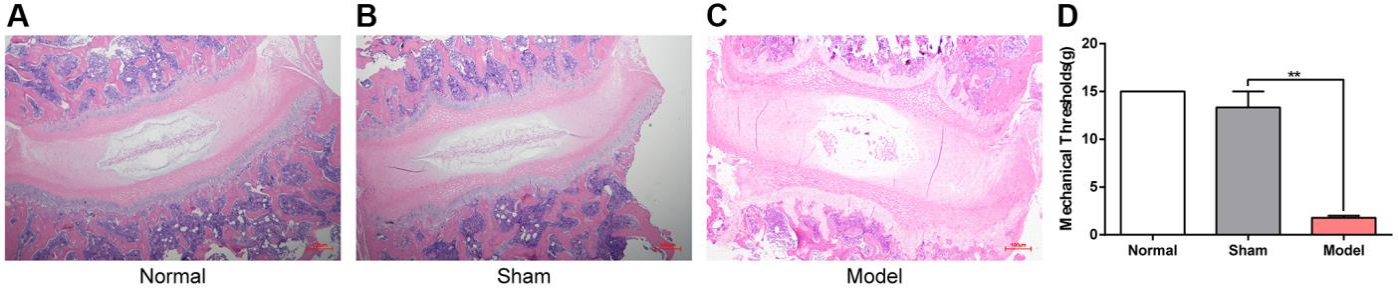
**Histomorphology of the cervical intervertebral disc and the neck-skin response thresholds to the mechanical stimuli 12 weeks after modeling.** (**A**–**C**) H&E staining of cervical intervertebral disc in different groups 12 weeks after modeling, note: Scale bar = 100 µm. (**D**) The neck-skin response thresholds to the mechanical stimuli applied by von Frey filaments in different groups 12 weeks after modeling, note: values are means ± SEMs, *n* = 3 per group, ^**^p < 0.01.

Hematoxylin and eosin (H&E) staining was carried out to detect the histopathological features of the cervical intervertebral disc by light microscopy. The intervertebral discs in the normal and sham groups had normal morphological and structural features, but those of model rats were altered, with enhanced ECM production and shrinkage and cluster aggregation of the NP (Figure 1A–1C). Meanwhile, model rats had substantially reduced neck-skin response thresholds to mechanical stimuli applied using von Frey filaments 12 weeks after operation (Figure 1D). These observations indicated a successful CS model establishment.

### Interventions and sample preparation

Twelve weeks post-modeling, treatments were administered. The normal, sham, and model groups underwent oral administration of pure water at 2 mL/day for 28 days. The M+Ms group was intragastrically administered celecoxib (Celebrex; Pfizer, USA) in pure water at 10 mg/kg/day for 28 days. The M+Ns group received pure water at 2 mL/day for 28 days and administered Ns.

Four points were selected as fixed intervention points, including the superior nuchal line (muscle insertion such as the trapezius, semispinalis capitis, and splenius capitis) and the internal superior angle of the scapula (muscle insertion of the levator scapulae). Additionally, 3–4 knot nodes of extensor/flexor muscle fibers around the cervical vertebra were chosen considering individual condition. Following disinfection, an Ns (HZ series: 0.4 × 40 mm, Beijing Outstanding Huayou Medical Instrument) was pierced to release the above points as described in a previous work [23]. After receiving the indicated treatment for 28 days, magnetic resonance imaging (MRI) examination of the cervical vertebra and neck pain-related behavior tests were performed before rats were humanely euthanized.

Next, C5/6 cervical intervertebral disc samples were obtained and submitted to fixation with 4% paraformaldehyde and 2.5% glutaraldehyde for transmission electron microscopy and histological assessment. C4/5 disc tissue samples were obtained and kept at −80°C for immunoblot and quantitative real-time PCR.

### von Frey test of mechanical allodynia

The pain-associated behavior of CS rats was assessed at 12 and 4 weeks after modeling and treatment, respectively, in a quiet environment at 25°C with low-intensity light from 9 AM to 6 PM. The animal’s neck and back were shaved with an HC1066 hair clipper (Philips, Amsterdam, Netherlands). Each rat was acclimatized to a black wire box for 30 min. Withdrawal responses to mechanical stimuli were examined with calibrated von Frey filaments applied from above the cage to the skin 2 mm away from the wound or the same area in control animals. The cutoff was 15 g, and the first filament evoking ≥3 responses (5 applications totally) was considered the threshold. In case of no response, 15 g was recorded. A response was defined as previously suggested [38].

### MRI

MRI examination was performed 4 weeks after treatment on a Bruker BioSpec 94/70USR scanner (Bruker BioSpin MRI GmbH, Ettlingen, Germany) with an animal surface coil to detect alterations in the central sagittal image of the cervical intervertebral disc on T2-weighted (T2W) scans. Disc degeneration levels were assessed based on the Pfirrmann MRI grading scale (Table 1). The image acquisition parameters were: repetition/echo time, 2500/33 ms; field of view, 40 × 40 mm; acquisition matrix, 256 × 256; number of slices, 5; slice thickness, 0.4 mm. MRI data were independently assessed by two experienced radiologists in a blinded manner.

**Table 1:** Pfirrmann MRI grading scale.

**Grade**	**Structure**	**Distinction of nucleus and annulus**	**Signal intensity**	**Height of Intervertebral disc**
**I**	Homogenous, bright white	Clear	Hyperintense, isointense to cerebrospinal fluid	Normal
**II**	Inhomogenous with or without horizontal bands	Clear	Hyperintense, isointense to cerebrospinal fluid	Normal
**III**	Inhomogenous, gray	Unclear	Intermediate	Normal to slightly decreased
**IV**	Inhomogenous, gray to black	Lost	Intermediate to hypointense	Normal to moderately decreased
**V**	Inhomogenous, black	Lost	Hypointense	Collapsed disc space

### H&E staining of intervertebral disks

Intervertebral disks from rats underwent fixation with 10% formalin, followed by a 2-week decalcification with EDTA. After deparaffinization and rehydration, the sections were submitted to H&E staining. A microscope was used for analysis, based on histological grading criteria for intervertebral discs (Table 2).

**Table 2:** Histological grading criteria of intervertebral disc based on staining.

**Histological nature**	**Grade**	**Histologic characteristics**
Morphology AF	1	Well-organized, half ring-shaped structure, collagen lamellae
	2	Partly ruptured AF; loss of half ring-shaped structure
	3	Completely ruptured AF; no intact half ring-shaped collagen lamellae
Cellularity NP	1	Normal cellularity; no cell clusters
	2	Mixed cellularity; normal pattern with some cell clusters
	3	Mainly clustered cellularity, chondroid nests present
NP matrix staining	1	Intense staining; blue stain dominates
	2	Reduced staining; mixture of blue and slight red staining
	3	Faint staining; increased red staining
Boundary AF and NP	1	Clear boundary between AF and NP tissue
	2	Boundary less clear; loss of annular-nuclear demarcation
	3	No distinguishable boundary between AF and NP tissue

### Ultrastructural assessment by electron microscopy

Each intervertebral disc was excised without the vertebral body for transmission electron microscopy (TEM). Then, intervertebral discs underwent a 2-h fixation with 2% glutaraldehyde, and phosphate-buffered saline (PBS) was used to remove any excess fixative. Next, fixation with 1% osmium tetrachloride was performed, and graded concentrations of ethyl alcohol were employed to dehydrate the specimens. The samples were then embedded in epoxy resin and underwent staining with toluidine blue. After locating target areas in these sections, ultrathin sections were obtained for double staining with uranyl acetate and lead citrate before TEM.

### Immunoblot

Total protein extraction from intervertebral disc samples utilized chilled RIPA buffer with phenylmethanesulfonyl fluoride (1 mM). Protein quantitation was carried out with the BCA Protein Assay Kit (P0012, Beyotime Biotechnology, Shanghai, China). Equal amounts of total protein (30 μg) were resolved by SDS-PAGE, with subsequent transfer onto a polyvinylidene fluoride (PVDF) membrane. After a 2-h blocking with 5% nonfat milk in TBST (20 mM Tris-HCl, pH 7.5, 150 mM NaCl, and 0.05% Tween-20) at ambient, overnight incubation was carried out with primary antibodies targeting SDF-1 (1:2500, ab9797, Abcam, UK), CXCR4 (1:100, ab124824, Abcam), Bcl-2 (1:500, ab196495, Abcam), Bax (1:1000, ab32503, Abcam), type II collagen (1:1000, ab188570, Abcam) and GAPDH (1:2000, E-AB-20059, Elabscience, Wuhan, China) at 4°C. Next, a 2-h incubation was performed with HRP-linked goat antirabbit IgG (H + L) secondary antibodies (1:5000, EF0002, SparkJade, Shandong, China) at ambient. The enhanced chemiluminescence kit was employed for detection, and quantitation used ImageJ.

### Quantitative real-time PCR

Total RNA extraction from cervical intervertebral disc employed the SPARKeasy Bone Tissue RNA Kit (SparkJade, AC1301, China) as directed by the manufacturer. RNA quantitation employed a Merinton^®^SMA4000 spectrophotometer (Merinton, Beijing, China) and reverse transcription employed a Reverse Transcription kit (SparkJade, AG0304, China) as instructed by the manufacturer. qPCR was carried out with 2 × SYBR Green qPCR Mix (SparkJade, AH0104, China). The 2^−ΔΔCt^ method was used for data analysis, with GAPDH as a reference gene. Table 3 describes the primers (Accurate Biotechnology, China) used for qPCR.

**Table 3:** Primers used in this study.

**Gene**	**Primer sequences**
SDF-1	Forward: GAGCCAACGTCAAACATCTGAA
	Reverse: ACTTGTTTAAGGCTTTGTCCAGGTA
CXCR4	Forward: AGTGACCCTCTGAGGCGTTTG
	Reverse: GAAGCAGGGTTCCTTGTTGGAGT
Bcl-2	Forward: GACTGAGTACCTGAACCGGCATC
	Reverse: CTGAGCAGCGTCTTCAGAGACA
Bax	Forward: GGCGATGAACTGGACAACAAC
	Reverse: CCACGGAAGAAGAAGACCTCTC
Collagen type II	Forward: AAGAGCAAGGAGAAGAAGCACAT
	Reverse: AGTGGACAGTAGACGGAGGAA
GAPDH	Forward: GGCACAGTCAAGGCTGAGAATG
	Reverse: ATGGTGGTGAAGACGCCAGTA

All sequences are in the 5′ to 3′ orientation.

### Immunohistochemistry for SDF-1, CXCR4, Bcl-2, Bax and Type II collagen detection in the intervertebral disc

Paraffin-embedded sections underwent dewaxing with xylene and hydration using an ethanol gradient. After deparaffinization and rehydration, incubation was performed with 3% hydrogen peroxide, followed by antigen retrieval with citrate buffer for 15 min at 92–98°C. Next, samples underwent overnight treatment at 4°C with antibodies targeting SDF-1 (1:250, ab9797), CXCR4 (1:500, ab124824), Bcl-2 (1:100, ab196495), Bax (1:250, ab32503) and collagen type II (1:200, ab34712), all from Abcam. After rinsing, a 1-h incubation was performed with horseradish peroxidase-linked secondary antibodies at ambient. The DAB kit (Zhongshan Golden Bridge Biotechnology, Beijing, China) was employed for development, followed by hematoxylin counterstaining. A BX60 microscope (Olympus, Tokyo, Japan) was utilized for imaging, and data analysis was performed with Image-Pro Plus.

### TdT-mediated dUTP nick-end labeling (TUNEL) staining

The TUNEL assay was performed to examine apoptosis in NPCs using the DAB (SA-HRP) TUNEL Cell Apoptosis Detection Kit (Servicebio, G1507, China). Paraffin-embedded sections underwent deparaffinization and rehydration with xylene and graded ethanol, respectively. After a 20-min treatment with proteinase K at ambient, specimens were incubated with 50 μL of TUNEL reaction mixture, shielded from light for 1 h at ambient. Next, a 30-min incubation was carried out with converter peroxidase (50 μL) away from light. After staining with 50 μL of 3,3′-diaminobenzidine for 10 min at ambient, hematoxylin counterstaining was performed for 3 min. Multiple high-power fields were randomly imaged with a microscope (Olympus) to determine the percentage of TUNEL-positive NPCs (yellowish-brown signals) relative to the total number of NPCs.

### Statistical analysis

SPSS 23.0 (SPSS, USA) and GraphPad Prism 8.0.1 (GraphPad, USA) were employed for data analysis. Data are mean ± standard error of the mean (SEM). Group pairs were compared by unpaired Student’s *t*-test, while multiple groups were compared by one-way analysis of variance (ANOVA) or two-way repeated-measures ANOVA, followed by post hoc Tukey test. The Kruskal–Wallis nonparametric test was performed to compare data with skewed distribution. *P* < 0.05 indicated statistical significance.

## RESULTS

### Effect of Ns on the histological features of the intervertebral disc

**Figure 2:**
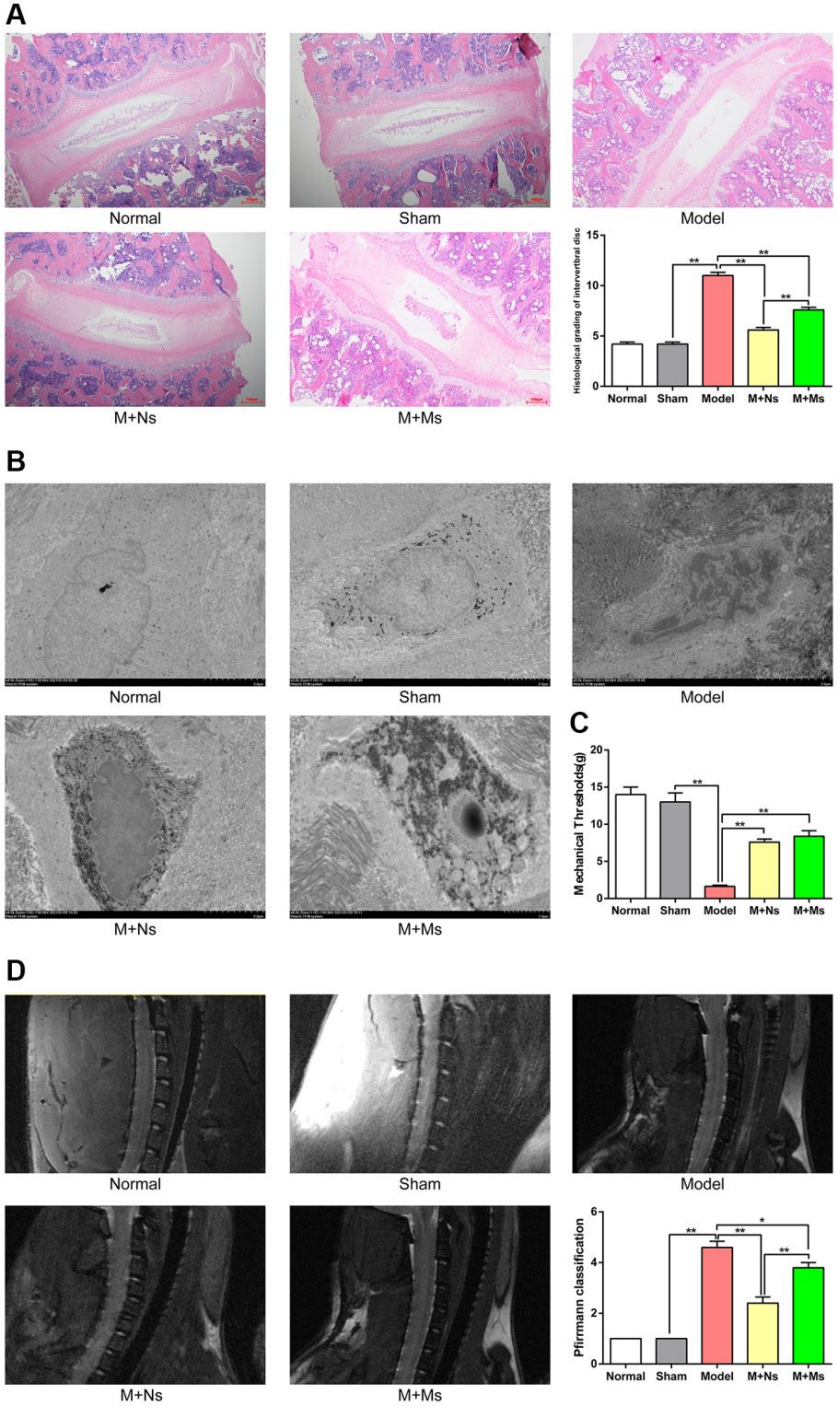
**The therapy of Ns affected intervertebral disc degeneration in the intervertebral disc of CS rats.** (**A**) H&E staining of cervical intervertebral disc in different groups 4 weeks after intervening, note: scale bar = 100 um. (**B**) Transmission electron microscope of the nucleus pulposus in different groups 4 weeks after intervening, note: scale bar = 2 um. (**C**) The neck-skin response thresholds to the mechanical stimuli applied by von Frey filaments in different groups 4 weeks after intervening. (**D**) Pfirrmann MRI Grades in different groups 4 weeks after intervening. Note: values are means ± SEMs, *n* = 5 per group, ^*^p < 0.05, ^**^p < 0.01.

Following 28 days of treatment, the collected cervical intervertebral disc samples underwent H&E staining and were imaged at 100×. As shown in Figure 2A, intervertebral discs in the normal control and sham groups exhibited regularly arranged annulus fibrosus and NP with a clearly delineated boundary between the annulus fibrosus and NP. The model groups, however, exhibited significant alterations, with scattered annulus fibrosus, clustered cellularity of the NP, and no distinguishable boundary between annulus fibrosus and NP. Intervertebral discs and annulus fibrosus in the M+Ns group were relatively regular, while slightly shrunken NP and visible cartilage endplates were detected. The M+Ms group had irregular intervertebral discs, with scattered annulus fibrosus and mixed cellularity, a normal appearance of some cell clusters of the NP, and a less clear boundary with loss of annular-nuclear demarcation.

Histological grading criteria for the intervertebral disc were compared among groups based on staining signals, reflecting disc degeneration degree. The values obtained were markedly lower in the normal and sham groups than in the remaining groups (*p* < 0.01). The model group showed elevated values in comparison with the M+Ns and M+Ms groups (*p* < 0.01), with the M+Ms group having elevated values compared with the M+Ns group (*p* < 0.01).

### Effect of Ns on NP ultrastructure

As shown in Figure 2B, after 28 days of treatment, cells in the normal and sham groups were round or oval, with a visible plasma membrane and a centrally placed nucleus with a smooth and whole nuclear membrane. Nuclei had an even distribution, with no overt heterochromatin. Furthermore, multiple cellular organelles were observed, e.g., the rough endoplasmic reticulum, mitochondria, matrix vesicles, and vacuoles. Cells in the model group exhibited irregular shapes, with an uneven nuclear membrane structure; heterochromatin had higher abundance compared with euchromatin, forming a “crest” that surrounded the nuclear membrane. The cytoplasm showed shrinkage, increased intranuclear electron density, and a decreased number of organelles. Mitochondria appeared unclear and cavitated, the rough endoplasmic reticulum showed overt swelling, the numbers of lysosomes and vacuoles increased, and lipid droplets were detected. Moreover, some cells around the acupoints of M+Ns treated rats were mainly oval or irregularly shaped. The plasma membrane was round, while the nuclear membrane was whole but slightly uneven. The intranuclei showed an even distribution, with heterochromatin molecules surrounding the nuclear membrane and organelles (e.g., the rough endoplasmic reticulum and mitochondria) showing mild swelling. However, there were some lysosomes and vacuoles. Cells from rats of the M+Ms group exhibited irregular shapes, an uneven nuclear membrane structure, increased heterochromatin, shrunken cytoplasm, fewer organelles, swollen rough endoplasmic reticulum and mitochondria, and thickened periplasmic compartment.

### Effect of Ns on the response threshold to mechanical stimuli induced by von Frey filaments

Neck-skin response thresholds non-significantly differed between the normal and sham groups at 4 weeks after intervention (*p* > 0.05). In comparison with sham animals, the model group exhibited reduced response threshold to mechanical stimuli (*p* < 0.01). Response thresholds to mechanical stimuli increased significantly (*p* < 0.01) in the M+Ns and M+Ms groups versus the model group, whereas the M+Ns and M+Ms groups had similar values (Figure 2C).

### Effect of Ns on Pfirrmann MRI grades

As shown in Figure 2D, MRI scans revealed the whole configuration of the cervical vertebra. In the normal control and sham groups, T2W images showed homogenous and bright white intervertebral disc structure, with clear boundaries of the nucleus and annulus, hyperintense signal intensity, and normal height. In the model group, the T2W sequence showed inhomogeneous and black intervertebral disc structure, with unclear boundaries of the nucleus and annulus, hypointense signal intensity, and collapsed height. In the M+Ns group, T2W scans showed inhomogeneous and gray intervertebral disc structure, with unclear boundaries of the nucleus and annulus, intermediate signal intensity, and slightly decreased height. In the M+Ms group, the T2W sequence showed inhomogeneous and gray to black intervertebral disc structure, with unclear boundaries of the nucleus and annulus, intermediate-to-hypointense signal intensity, and moderately decreased height.

We compared Pfirrmann classes among groups according to cervical intervertebral degeneration level. The grades were markedly lower in the normal and sham groups in comparison with the model group (*p* < 0.01). Meanwhile, the model group had higher grades compared with the M+Ns and M+Ms groups (*p* < 0.01), with the M+Ms group showing higher values versus the M+Ns group (*p* < 0.01).

### Ns therapy regulates SDF-1, CXCR4, Bcl-2, and Bax in the intervertebral disc of CS rats

**Figure 3:**
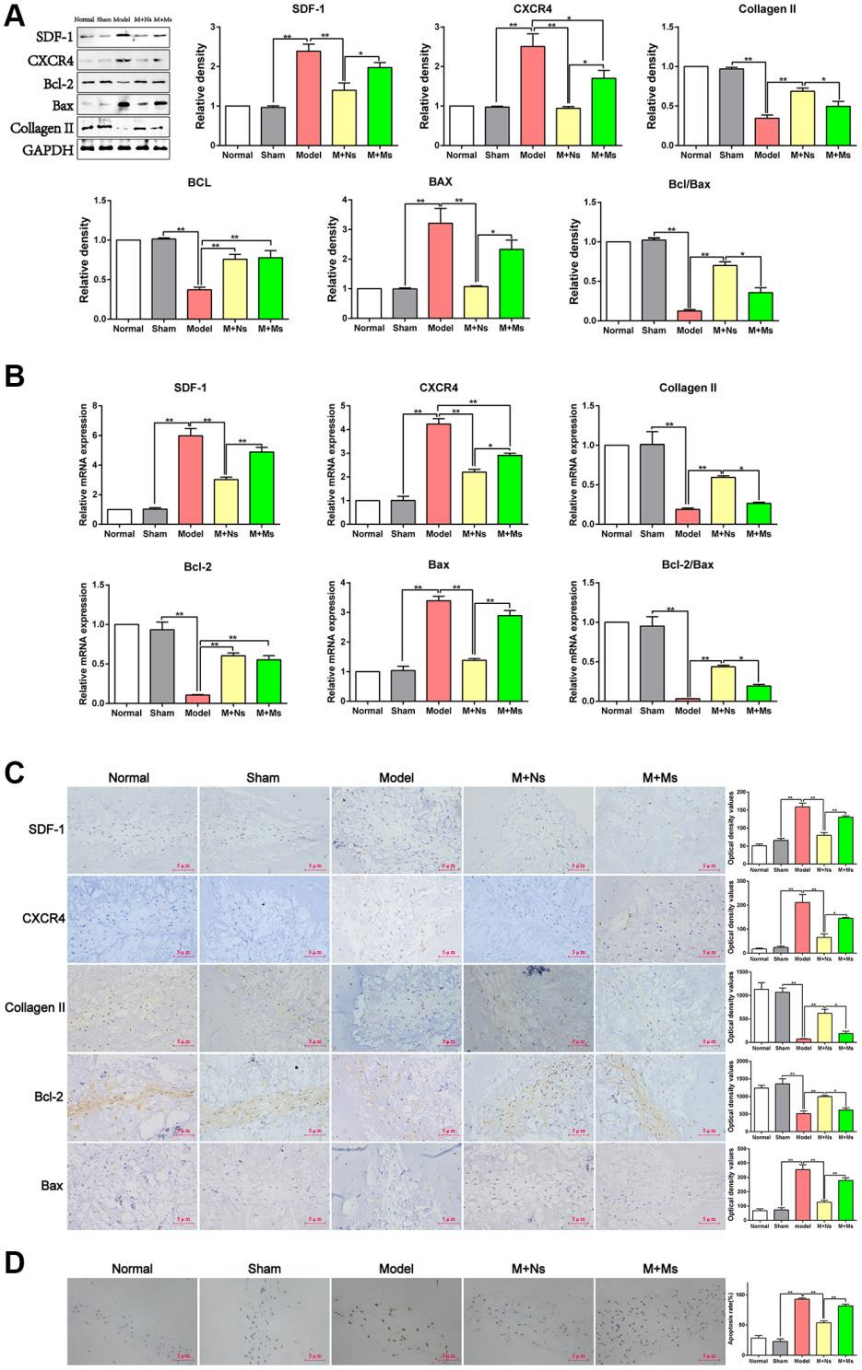
**The therapy of Ns affected apoptosis of nucleus pulposus cells mediated by specifically regulating SDF-1/CXCR4 signal axis in the intervertebral disc of CS rats.** (**A**) Western blot assay of SDF-1/CXCR4, Bcl-2/Bax and type II collagen in different groups 4 weeks after intervening. (**B**) Real-time PCR analysis of SDF-1/CXCR4, Bcl-2/Bax and type II collagen in different groups 4 weeks after intervening. (**C**) Immunohistochemical staining and optical density value of SDF-1/CXCR4, Bcl-2/Bax and type II collagen in different groups 4 weeks after intervening. (**D**) TUNEL staining to confirm the effect of 4 weeks of intervention on apoptosis of nucleus pulposus cells in different groups. Note: scale bar = 5 um, values are means ± SEMs, *n* = 5 per group, ^*^p < 0.05, ^**^p < 0.01.

Whether these treatments affect apoptosis in NPCs by regulating SDF-1/CXCR4, Bcl-2/Bax, and type II collagen was examined next. Western blotting revealed markedly increased SDF-1, CXCR4, and Bax protein amounts (*p* < 0.01) in the model group versus sham rats, while these proteins were starkly downregulated (*p* < 0.05) in the M+Ns and M+Ms groups relative to the model group. However, the mRNA expression levels of only CXCR4 were substantially decreased (*p* < 0.01) in the M+Ms group versus the model group. A comparison of both therapies revealed SDF-1, CXCR4, and Bax were markedly upregulated in the M+Ms group (*p* < 0.05) versus the M+Ns group; therefore, the Ns therapy showed a better tendency to inhibit SDF-1, CXCR4, and Bax upregulation. Conversely, Bcl-2, Bcl-2/Bax, and type II collagen expression levels starkly decreased (*p* < 0.01) in the model group versus sham animals, while the M+Ns group showed remarkably increased levels versus the model group (*p* < 0.01). Most notably, Bcl-2/Bax and type II collagen expression levels were similar in the M+Ms and model groups (*p* > 0.05). Comparing both therapies, starkly reduced Bcl-2/Bax and type II collagen expression levels were detected for the M+Ms group in comparison with the M+Ns group (*p* < 0.05), while Bcl-2 expression levels did not differ between the two groups. Therefore, the Ns therapy could better inhibit the downregulation of Bcl-2, Bcl-2/Bax, and type II collagen (Figure 3A).

Quantitative real-time PCR yielded similar results. SDF-1, CXCR4, and Bax mRNA amounts were markedly increased (*p* < 0.01) in the model group versus sham animals, while the M+Ns group had starkly decreased amounts versus the model group (*p* < 0.01). However, the mRNA amounts of only CXCR4 were decreased significantly (*p* < 0.01) in the M+Ms group compared with the model group. Comparing the M+Ns and M+Ms groups, SDF-1, CXCR4, and Bax mRNA amounts were decreased in the M+Ns group (*p* < 0.05). Thus, the Ns therapy could better inhibit the increases of SDF-1, CXCR4, and Bax mRNA amounts. Conversely, Bcl-2, Bcl-2/Bax, and type II collagen mRNA amounts were starkly decreased (*p* < 0.01) in the model group versus sham animals, while the M+Ns group had remarkably increased amounts versus the model group (*p* < 0.01). However, Bcl-2/Bax and type II collagen mRNA amounts were similar in the M+Ms and model groups (*p* > 0.05). Comparing both therapies, Bcl-2/Bax and type II collagen mRNA amounts were starkly reduced in the M+Ms group versus the M+Ns group (*p* < 0.05), whereas the mRNA expression levels of Bcl-2 did not differ between the two groups. Therefore, the Ns therapy could better inhibit the decreases of Bcl-2, Bcl-2/Bax, and type II collagen mRNA levels (Figure 3B).

Immunohistochemistry staining further confirmed these findings. A comparison of the M+Ns and model groups corroborated western blot and quantitative real-time PCR data (Figure 3C). Specifically, SDF-1, CXR4, and Bax expression levels were markedly decreased in the M+Ns group compared with the model group (*p* < 0.01). Meanwhile, SDF-1, CXR4, and Bax expression levels were starkly higher in the M+Ms group versus the M+Ns group (*p* < 0.05). In contrast, Bcl-2 and type II collagen expression levels were markedly higher in the M+Ns group in comparison with the model group (*p* < 0.01) and remarkably reduced in the M+Ms group versus the M+Ns group (*p* < 0.05). These results suggested that the Ns therapy relieved apoptosis in the intervertebral disc by suppressing the SDF-1/CXCR4 axis in the NPCs of CS rats.

### Effect of Ns on TUNEL staining in NPCs

To further evaluate Ns’ effect on SDF-1/CXCR4 axis-mediated inhibition of apoptosis in NPCs, TUNEL staining was performed. As shown in Figure 3D, TUNEL staining revealed markedly elevated apoptotic rate for NPCs in the model group in comparison with sham animals (*p* < 0.01), while this rate remarkably decreased (*p* < 0.01) in the M+Ns group versus the model group. However, similar apoptotic rates for NPCs (*p* > 0.05) were detected in the M+Ms and model groups. Comparing both therapies, NPC apoptosis was substantially enhanced in the M+Ms group versus the M+Ns group (*p* < 0.01); therefore, the Ns therapy could better reduce the increase in the apoptotic rate of NPCs.

## DISCUSSION

CS is a syndrome characterized by cervical disc degeneration and secondary changes resulting from the mechanical imbalance of the cervical spine, involving surrounding tissue structures and inducing clinical manifestations. The etiology and pathogenesis of CS are complex, with evidence indicating that cervical disc degeneration is the initiating factor in the development of CS, and NPC apoptosis is a key event leading to cervical disc degeneration [39–42]. This study showed that CS induced IVDD and NPC apoptosis and increased neck pain; in addition, Ns therapy alleviated neck pain, mitigated IVDD, and reduced NPC apoptosis. These effects may be mediated by the SDF-1/CXCR4 axis in the intervertebral disc of CS rats.

CS rat models were induced by cervical static–dynamic imbalance for 12 weeks. As shown above, Ns therapy reduced the histological grade of the cervical disc, which is a direct reflection of cervical disc degeneration [43]. It also rescued the cervical disc degeneration detected by MRI and scanning electron microscopy from macro- and micro-perspectives, respectively. Additionally, Ns suppressed the elevated Bax levels and reduced Bcl-2 levels, which are biomarkers of apoptosis in NPCs, thereby increasing the Bcl-2/Bax ratio. We also found that SDF-1/CXCR4 signaling was specifically activated in the cervical disc of CS rats, probably activating downstream pathways in the cervical disc, including a decrease in type II collagen levels in the ECM and changes in the levels of related apoptotic factors, ultimately increasing apoptosis in NPCs and enhancing the degeneration of the cervical disc. Ns therapy could improve the abnormal apoptosis of NPCs by regulating the SDF-1/CXCR4 signaling axis.

Cervical static–dynamic imbalance represents a broadly employed technique for CS model establishment. By partially removing the posterior muscles and ligaments from the cervical vertebrae, mechanical imbalance of the cervical spine is induced, promoting the onset of CS caused by long-term head lowering in humans. Intervertebral discs are rich in type II collagen, and depletion of this protein is considered a biomarker of IVDD. Bcl-2, which inhibits apoptotic cell death, and Bax, which induces apoptosis, are key proteins in cell apoptosis, and the Bcl-2/Bax ratio in cells is an essential determinant of apoptosis [44]. Therefore, Bcl/Bax and the TUNEL assay were utilized to assess apoptosis in NPCs. The current study showed that cervical static–dynamic imbalance induced mechanical allodynia in the neck of rats, as measured by the von Frey test, and histological grade increased in the intervertebral disc, as detected by H&E staining. Apoptosis increased in CS rats, consistent with the observed changes in NPC apoptosis in patients with CS in a previous study. According to a systematic review, apoptosis of NPCs is associated with radiographic and painful CS [45, 46]. These findings indicate that rats with static–dynamic imbalance for 12 weeks may show CS-like intervertebral disc lesions resembling those detected in humans.

Imaging features of CS mainly include cervical lordosis or straightness and cervical disc degeneration. The muscles and ligaments around the cervical spine maintain biomechanical balance [47]. After CS occurrence, the muscles behind the cervical spine remain in a tense state for a prolonged time, and the surrounding tendons form hard knots or inflammatory adhesions. These reaction points further increase the stress on the cervical intervertebral disc and accelerate its degeneration [48–50]. Therefore, we selected the tendon node around the cervical spine as the Ns intervention point. The mechanical balance of the cervical intervertebral disc was restored via adjustment of the mechanical function of the cervical spine. Applying MRI, SEM, and mechanical pain analyses, the present study found that both Ns therapy and Ms therapy reduced neck pain and delayed IVDD. Although both Ns and Ms therapies significantly delayed disc degeneration progression, the pathological alterations of the disc were not entirely reversed. Our findings indicate that enhanced apoptosis in NPCs could be prevented by Ns therapy, which corroborates previous data suggesting an association of apoptotic rate in NPCs with IVDD.

SDF-1/CXCR4 signaling substantially contributes to apoptosis in intervertebral disc NPCs. Research has confirmed SDF-1 and CXCR4 show elevated expression in degenerated intervertebral discs, with upregulated SDF-1 and CXCR4 enhancing apoptosis in NPCs. SDF-1/CXCR4 signaling regulates various pathological processes in IVDD, including inflammation, mechanical functions, and angiogenesis [30–37]. However, whether Ns therapy inhibits the activation of SDF-1/CXCR4 signaling in NPCs and affects cell apoptosis is currently unknown. The present study demonstrated a pivotal role for SDF-1/CXCR4 signaling in NPC apoptosis in CS rats. As shown above, CS remarkably elevated SDF-1 and CXCR4 mRNA and protein amounts, while Ns therapy inhibited these increases in the cervical intervertebral disc.

CS-induced neck pain is an important clinical manifestation of disease progression, seriously affecting the quality of life and leading to other complications. Consequently, the currently available treatment strategies for CS mostly aim to alleviate neck pain and subsequently mitigate the progression of IVDD. The above data confirmed Ns and celecoxib effectively induce analgesia in rats with experimental CS, although both treatments may have distinct underpinning mechanisms. Celecoxib is a selective NSAID with a reduced incidence of gastrointestinal side effects compared with other NSAIDs and is widely used in clinical practice to alleviate neck pain [15]. Ns is a nonpharmaceutical and common alternative treatment that exerts analgesic effects in rats with CS by alleviating the abnormal tension on cervical muscles and regulating the abnormal mechanical properties of the cervical intervertebral disc, ultimately alleviating pain. Furthermore, this work demonstrated Ns treatment significantly improved apoptosis in NPCs compared with celecoxib treatment based on related indicators. Ns and celecoxib both alleviated NPC apoptosis in CS rats, although likely employing different mechanisms. We hypothesize that combining Ns with celecoxib or other NSAIDs may result in synergistic effects in CS treatment, which deserves further investigation.

In summary, Ns therapy mitigated cervical disc degeneration by decreasing NPC apoptosis in CS rats; this inhibition might be mediated by SDF-1/CXCR4 signaling. These results provide a more solid theoretical foundation for the use of Ns in CS therapy.
